# Detection of measles virus in Bulgaria from to 2018

**DOI:** 10.3325/cmj.2022.63.475

**Published:** 2022-10

**Authors:** Stefka Krumova, Sabine Santibanez, Ivona Andonova, Radostina Stefanova, Annette Mankertz, Todor Kantardjiev

**Affiliations:** 1 Department of Virology, National Centre of Infectious and Parasitic Diseases, Sofia, Bulgaria; 2 National Reference Center for Measles, Mumps, Rubella, Robert Koch Institute, Berlin, Germany; 3 National Centre of Infectious and Parasitic Diseases, Sofia, Bulgaria

## Abstract

**Aim:**

To determine the circulation patterns of measles virus in Bulgaria from 2012 to 2018 after a large measles outbreak in the country (2009-2011).

**Methods:**

Three types of clinical material were collected: serum samples, urine samples, and nasal swabs. Enzyme-linked immunosorbent assay (ELISA) was used to detect specific viral immunoglobulin (Ig) M/IgG antibodies. Viral RNA was extracted from all urine and nasal swabs.

**Results:**

In the investigated period, 102 patients were confirmed to have measles (age range: two months to 55 years). A total of 101 samples (99%) were measles-IgM positive. Most of them were detected in 2017 (73%, 74/101), when a measles outbreak in the country was reported. The majority of patients were unvaccinated children aged under 13 months. Out of 101 measles serum samples confirmed by ELISA, 18 (20.45%) were measles-IgG positive and 15 (17.05%) were borderline. Thirty-three positive PCR products were sequenced and genotyped. In 2013, 2016, 2017, and 2018, three different measles viral genotypes were detected: D8, H1, and B3. Most patients were unvaccinated or insufficiently vaccinated.

**Conclusion:**

Preventive measures are indispensable to limit the infection in different regions of Bulgaria and its spread to other countries. As vaccination coverage against measles and other vaccine-preventable infections, including SARS-Co2, is low, it is necessary to perform molecular identification of viruses to monitor their circulation and pathogenicity.

Measles remains the main cause of morbidity and mortality in childhood globally. Due to the high congestive index, the presence of severe, debilitating complications, high incidence, and significant lethality, the virus imposes a considerable health and socioeconomic burden (1-3). The disease is preventable by vaccination (with monovalent vaccine or multivalent vaccines protecting also against rubella, mumps, and chickenpox) in childhood and adolescence, which provides lifelong immunity to most recipients (4).

The measles virus (MV) is serologically monotypic, and 24 measles genotypes (A – H) have been identified. All used vaccine strains (Moraten, Edmonston, Zagreb) are from the MV genotype A (4,5). Since 2011, 8 genotypes have been circulating globally: B2, B3, D4, D8, D9, D11, G3, and H1.

The elimination of vaccine-preventable diseases as measles and rubella is a priority of the World Health Organization (WHO) strategic plan. However, there are a number of challenges in the process of the verification of elimination – maintaining high vaccine coverage and effective national surveillance systems, wild virus circulation, expanding epidemic outbreaks, and other.

According to the WHO guidelines, the laboratory detection of MV is based on the isolation of viral RNA from appropriate clinical samples, on demonstrating in serum or saliva a specific antibody response against the MV characteristic of acute infection, or on MV antigen detection by direct fluorescent antibody assay (DFA) (6). The main approaches for national confirmation of measles cases (according to laboratory capacity) are immunoenzymatic approach and molecular-biological approach with reverse transcriptase polymerase chain reaction (PCR). The National Reference Laboratory in Bulgaria applies both analyses in order to avoid false-negative results. The combined laboratory diagnostic approach and epidemiological surveillance are the main criteria for confirming the elimination of MV spread. The molecular genetic analysis during the elimination period aims to obtain genetic information from each chain of viral transmission. The elimination is confirmed by the absence of endemic genotypes in the country for one year. On the other hand, only sequence analysis can distinguish between vaccine reactions and infections with the wild-type virus (7,8). In Bulgaria, national case-based notification of measles started in 2004. In 2005, the European Union case definition and case classification were adopted for surveillance purposes (*http://mmr.gateway.bg/en/)* (9,10).

In the period 2002-2004, no local cases of measles were reported in Bulgaria, and for three consecutive years the incidence was 0.0%. Between 2005 and 2008, a total of six cases of measles were registered. Four of these were imported from other countries and two were import-related cases. In 2009-2011, despite the high immunization coverage ( ~ 95%), one of the largest outbreaks of MV in Europe was reported, with 24 365 diseases (324%) and 24 deaths (mortality 0.3%, lethality 0.1%) (11,12). The aim of present study was to determine the circulation patterns of MV in Bulgaria in the period 2012-2018 after the large measles outbreak 2009-2011.

## Patients and methods

### Clinical samples

Data were collected retrospectively from web-based National Measles Surveillance System (NMSS) in Bulgaria. Between 2012 and 2018, 102 patients were confirmed to have measles (age range two months to 55 years). Three types of clinical material were collected: serum samples, urine samples, and nasal swabs. All the samples were collected as part of the measles/rubella surveillance system in Bulgaria with the cooperation of regional hospitals and regional health agencies in the country.

As the only WHO accredited-laboratory in the country, the National Reference Laboratory for Measles, Mumps, and Rubella at the National Center of Infectious and Parasitic Diseases (NCIPD), Sofia performs combined serological and molecular biological tests for each suspected measles case by using Measles ELISA IgM/IgG, Measles ELISA Avidity test, and RT-PCR analysis. According to the virus specificity and the recommendations of a number of authors, measles detection is performed in serum samples, urine samples, and nasal swabs (13-15). According to MV case definition, cases with positive anti-measles ELISA IgM and/or RT-PCR results are considered as laboratory confirmed.

### Enzyme-linked immunosorbent assay (ELISA)

Blood samples were taken by venipuncture during patients’ hospital stay. Blood was centrifuged at 1000 g for 10 min, and serum was aliquoted and frozen at -20 °C until analyses were performed.

All serum samples were tested for the presence of anti-measles IgM and IgG antibodies. Anti-measles IgG-positive specimens were tested with avidity test by using a commercial indirect ELISA kits (Siemens ELISA IgM [Siemens Healthcare Diagnostics Products GmbH, Marburg, Germany], Euroimmun, ELISA IgM/IgG and Euroimmun, ELISA Avidity IgG kits [Euroimmun, Lübeck, Germany]). The results were analyzed according to the manufacturer's recommendations and interpreted as positive, negative, or ambiguous; and for measles IgG-positive samples, as having low or high avidity.

### RNA extraction and polymerase chain reaction (PCR)

MV RNA was extracted from urine samples and nasal swabs with Qiagen Viral RNA test kits (QIAGEN GmbH, Hilden, Germany). A one-step reverse transcription PCR (RT-PCR) for amplification of MV nucleoprotein (N) gene with primer pairs MeV 214 and MeV 216 was performed (7,16). Each PCR included negative and positive controls. MV-positive samples were subjected to subsequent sequencing and phylogenetic analysis.

### Electrophoresis assays

MV PCR products (634 nt) were analyzed on 2% agarose gels stained with ethidium bromide.

### Phylogenetic analysis

Phylogenetic analyses were carried out with Molecular Evolutionary Genetics Analysis (MEGA) software, version 7 (17), the Kimura 2-parameter model, and the neighbor-joining algorithm. For genotyping, phylogenetic trees were constructed based on 450 nucleotides that encode the carboxy-terminal region of the nucleoprotein (N–450) of MV. Genotyping and phylogenetic analysis were performed at the WHO European Regional Reference Laboratory for Measles and Rubella, Robert Koch Institute, Berlin, Germany and the NCIPD in Sofia.

## Results

In the seven-year period from 2012 to 2018, 101 serum samples, 23 urine samples, and 68 nasal swab specimens from 102 patients were confirmed to be measles-positive. In 92 patients, two types of clinical specimens (serum samples and urine or nasal swab) were collected, and 10 were confirmed only by ELISA in serum samples.

### Serological analysis

**Figure 1:**
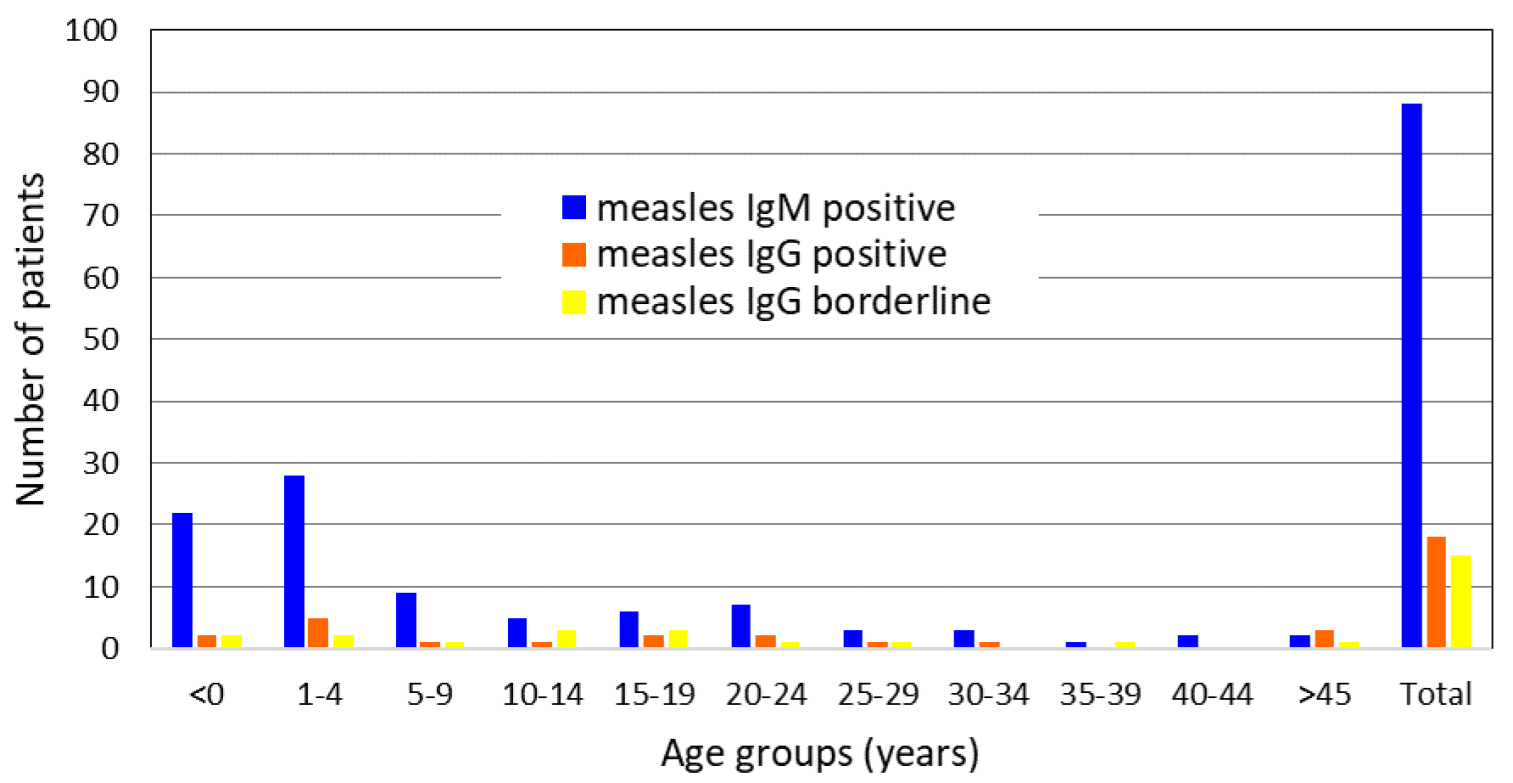
The number of patients confirmed with having measles-specific immunoglobulin M (IgM) and IgG according to age groups, 2012-2018 (n = 101).

A total of 101 of 102 sera (99%) were measles-IgM positive. The confirmed cases were from seven Bulgarian regions, the most affected being Plovdiv (n = 50), Pazardjik (n = 20), and Sofia regions (n = 15) (Table 1). More than half of the samples (73%, 74/101) were detected in 2017, when a measles outbreak occurred in the country. In 2014 and 2015, there were zero positive cases. In 2012 and 2016, only sporadic cases with one laboratory confirmed case were observed. The case in 2016 was confirmed only by RT-PCR in urine (Table 1). The majority of positive cases were among unvaccinated children aged under 13 months (22/102, 22%) and in the age group 1-4 years (28/102, 27%). Adults older than 40 years were least affected (4/102, 4%) (Figure 1).

**Table 1:** The number of laboratory-confirmed measles virus (MV) cases in the National Reference Laboratory and detected MV genotypes in Bulgaria by regions and years

Year	Region	Number of laboratory confirmed measles cases	Detected measles genotype	Country of importation
2012	Stara Zagora	1	ND	Poland
2013	Sofia	11	D8, “*D8-Frankfurt-Main*”	Germany
	Razgrad	1	D8, “*D8-Frankfurt-Main*”	Germany
	Sliven	1	D8, “*D8-Frankfurt-Main*”	Turkey
2016	Stara Zagora	1	H1	ND
2017	Plovdiv	50	B3, *MVs/Dublin.IRL/08.16*	Germany Romania
	Pazardjik	20	B3	Germany
	Montana	4	B3, *MVs/Dublin.IRL/08.16*	Romania
2018	Burgas	8	D8	Ukraine
	Lovech	1	ND	ND
	Sofia	4	B3, *MVs/Dublin.IRL/08.16*	Italy

*ND – no data.

**Figure 2:**
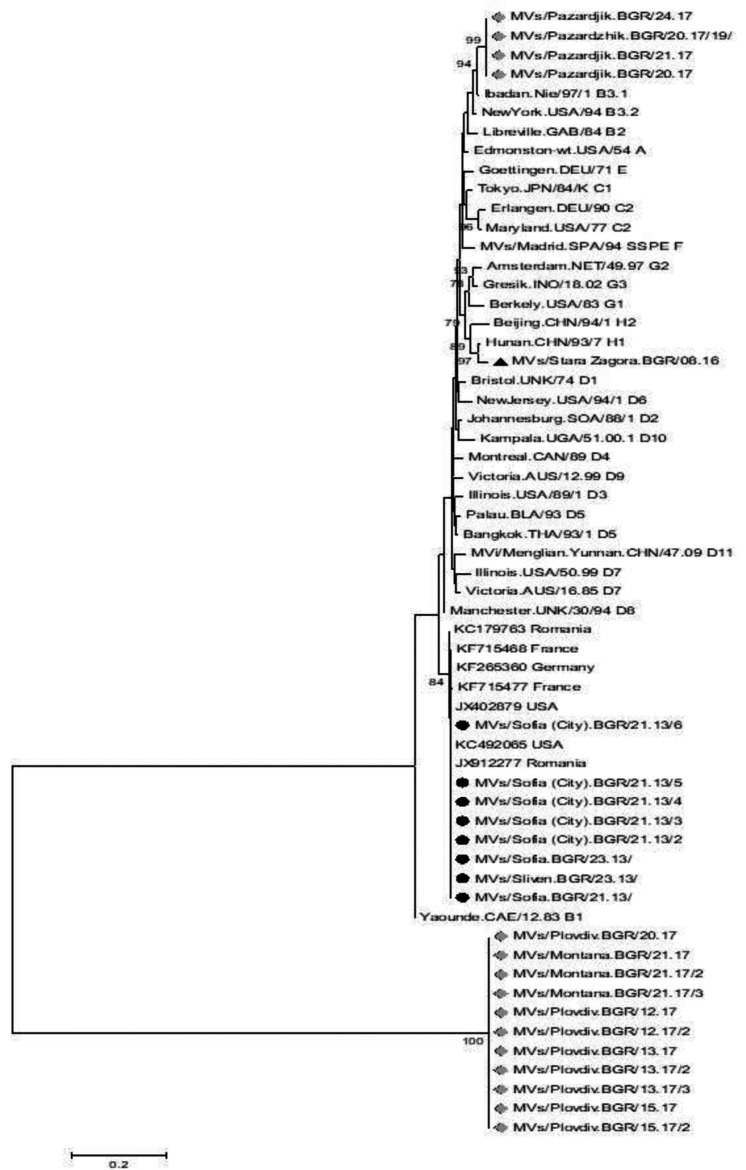
Phylogenetic tree based on a 450-nt region of the N gene of measles virus created using the Kimura 2-parameter model and the neighbor-joining algorithm of MEGA 5.05. Only bootstrap values ≥70% (1000 replicates) are shown. Bulgarian sequences are named based on the location, epidemiological week, and year. The sequences obtained during the present study are marked with black dots, and reference sequences are defined by accession number, name, and genotype. Bulgarian sequences from the period 2013-2017 are noted according to the year (2013: black circle, 2016: black triangle, 2017: gray rhombus). In the GenBank, we found identical strains from other countries, and reference sequences were determined by accession number, name and genotype.

Out of 101 ELISA-confirmed serum samples, 18 were IgG positive and 15 were borderline samples (Figure 2). In IgG-positive patients, measles avidity index was determined. A high avidity index was reported in 9/18 (50%) samples; low in 5/18 (27.78%); and borderline in 4/18 (22.22%).

### Molecular analysis

Measles RNA was analyzed with RT-PCR in 91 samples (23 urine samples and 68 nasal swabs). Thirty-three positive PCR products were sequenced and genotyped. In 2013, 2016, 2017, and 2018, three different viral genotypes were detected: D8, H1, and B3. An imported infection was confirmed on the basis of epidemiological information and genotyping.

The measles cases detected in 2013 were identified as imported from Germany or import-related. Local outbreaks occurred in three regions – Sofia (local family outbreak), Razgrad, and Sliven. The majority of the patients were unvaccinated or incompletely vaccinated individuals from the Roma population. The index case from Sofia was a 31-year-old unvaccinated man who arrived from Germany, where he was in contact with a Turkish woman who had a rash. Measles cases from the other regions were also imported from Germany or Turkey. The measles genotype in all three regions was D8, variant “*D8-Frankfurt-Main,*” circulating in Germany since February 2013 (Figure 2).

In 2016, there was one confirmed measles case in Bulgaria (H1 genotype). The patient was a 24-year-old woman with conjunctivitis and incomplete vaccination (1 dose of MMR) from the city of Stara Zagora. The woman had not traveled abroad, but she worked as a waitress at a roadside restaurant serving international drivers (Figure 2).

In the period 2013-2018, the highest number of registered and laboratory confirmed measles cases in Bulgaria was observed in 2017. The outbreak occurred in three regions (Plovdiv, Pazardjik, and Montana). The epidemiological surveillance showed a probable measles import from Germany, Romania, and Italy, and nosocomial transmission. Genotyping of positive PCR products confirmed the measles genotype B3, Strain MVs/Dublin.IRL/08.16 (for Plovdiv, Pazardjik, and Montana) (Figure 2).

In 2018, 13 MV cases were confirmed: eight in Burgas, four in Sofia, and one in Lovech. The patients were Ukrainian citizens on seasonal work on the Black Sea and Bulgarian citizens living in Italy, the United Kingdom, and Greece. In all cases, the clinical symptoms occurred within one week of the patients’ arrival in Bulgaria. B3 and D8 genotypes were detected.

## Discussion

During the investigated period, one measles case was registered in the country in 2012 and 2016, and limited epidemic outbreaks were reported in 2013 and 2017. Infected persons were mainly from the Roma population, and cases were related to imports from other European countries.

In the period 2013-2018, each confirmed measles case in Bulgaria was classified according to the WHO definition as an imported case (the patient arrived from a country with a proven MV circulation and could have been infected there 7-18 days before the onset of rash) or an import-related case (locally infected case as part of a proven chain of transmission of imported cases in the country; import-related cases persist for no longer than 12 months). The laboratory and epidemiological data and surveillance categorize Bulgaria as a country in the phase of measles elimination.

Even in the first decades of the 21st century in Europe, large measles outbreaks with over 15 000 patients were reported in Germany, Romania, Italy, France, and several Balkan countries. The measles incidence in Europe for 2016 was 5133 cases in 34 countries, and for the first half of 2017 – 9386 cases in 40 countries (18-21). In 2017, measles outbreaks occurred in Romania, Italy, Greece, and Germany, with 5560, 5004, 967, and 929 cases, respectively (22). A total of 37% of the reported cases were adults aged ≥20 years. In the same period, 179 measles deaths were reported, more of them during 2017-2018 in Romania, Ukraine, Serbia, and Italy. The most predominant MV genotypes detected were D4, D8 and B3 (23).

Major challenges in the elimination process remain maintaining high vaccination-coverage among risk groups and reducing the incidence in young children and infants who are under the vaccination age (24). In this study, the population most affected by measles were unvaccinated children under one year.

In the period 2009-2017, estimated EU coverage with the first MMR dose was 93%-95%, and the coverage with the second dose increased from 73% to 90% (25). The low vaccination coverage in Bulgaria (91%-93% MMR1 and 87%-91% MMR2) is a risk factor for importation and spread of measles in the country. A combined approach of serological and molecular confirmation of each case is important in the process of elimination (26). Molecular studies provide information about the circulation of measles genotypes in the world, predominance of one genotype, and the disappearance of another. From 2012 to 2017, three major measles genotypes circulated in Europe – D4 (2009-2013), D8 (2013-2018), and B3 (2014-2018) (8,23). These genotypes were mainly registered in Bulgaria in the investigated period.

The current study shows that the main routes of the import of MV into Bulgaria were Balkan countries and central European countries. All reported cases were laboratory- and epidemiologically confirmed, and most of the patients were unvaccinated or insufficiently vaccinated. In the same period (2012-2018), Germany, Romania, and Italy registered the most cases in Europe and had the potential to export the virus.

Widespread tourism and migrations, along with the high contagiousness of the virus, facilitate the spread of measles among European countries. Investigating the molecular epidemiology of measles is important for building a map of genotypic viral circulation and determining the aggressiveness and dominance of certain viral genotypes. The main limitation of the study is that one-third of the positive samples were sequenced. However, they provided data on the overall distribution of measles genotypes in the country, as the samples were from all regions of the country and all chains of transmission.

In an era of emerging viruses and the covid-19 pandemic, vaccine-preventable infections continue to be a problem in modern society. Despite the presence of a highly effective vaccine, MV remains a pathogen of social and economic importance. The presence of endemic regions enables the MV spread through travel and migrations. It is important to take immediate preventive measures to limit the infection in different regions of the country and to perform molecular identification of viruses in order to monitor their circulation and pathogenicity.
